# Adolescents' and children's expectations of fairness and bias in the classroom

**DOI:** 10.1111/jora.70236

**Published:** 2026-07-19

**Authors:** Elise M. Kaufman, Marley B. Forbes, Melanie Killen

**Affiliations:** ^1^ Department of Human Development and Quantitative Methodology University of Maryland, College Park College Park Maryland USA

**Keywords:** classroom racial bias, expectations of bias, social reasoning

## Abstract

This study examined U.S. youth (*N* = 303, 51% female, 47.5% Black/African American, 36.9% White/European American, 15.5% Asian American/Pacific Islander) expectations of racial bias in an academic context. Children (*n =* 172, ages 8–11, *M*_age_ = 10.34) and adolescents (*n =* 131, ages 12–14, *M*_age_ = 13.09) viewed vignettes in which a teacher or peer preferred one racial group (Asian, Black, or White) for academic recognition. Participants provided their predictions about the future biased behavior of the teacher and peer characters before and after the characters received a message promoting fairness. Findings indicated that adolescents, more so than children, viewed both teacher and peer racial bias as likely to persist. On average, participants expected teacher racial bias to persist regardless of which racial group they saw the teacher prefer. When predicting a peer's racial bias, youth predicted continued preferential treatment for Asian students to be less likely than preferential treatment for Black or White students. Participants who reported being in the racial‐ethnic minority at their own school expected that after the biased teacher or peer received a message promoting fairness, the characters would be less likely to show the same bias. Advancing knowledge about youth's expectations of whether a classroom racial bias is likely to continue or to change has important implications for understanding young people's motivations for challenging and resisting bias and social inequalities at school.

## INTRODUCTION

In the school context, informal academic recognition such as having one's work displayed in the classroom is one way teachers may communicate their perceptions of students' academic ability (Gibson & Hedley, 2025; Harris et al., 2020). Seeing one's academic work displayed in the classroom has been linked with higher self‐esteem and academic motivation among elementary‐aged children (Maxwell & Chmielewski, 2008), though this is underexplored among adolescents. Yet, inequalities in academic recognition based on a number of factors often exist in the classroom for both children and adolescents (İnan‐Kaya & Rubie‐Davies, 2021; Peterson et al., 2016; Staats, 2016). Racial‐ethnic minority students report experiencing bias even in schools in which teachers and students espouse color‐blind attitudes (Davis et al., 2022). This is important in the case of academic recognition because teacher underestimation of students' academic ability early in high school is associated with students' lower *self‐*assessment of academic ability and with lower future academic performance (Cherng, 2017). This study examined children's and adolescents' expectations and reasoning about racial bias in academic recognition by investigating whether youth perceive teacher and peer racial bias in the classroom as likely to change.

### Theoretical framework

This study draws from the social reasoning developmental (SRD) model (Rutland et al., 2010), which posits that in contexts of social inequality, individuals consider moral concerns (e.g., recognizing unfair bias), group identity concerns (e.g., race or ethnicity preferences), as well as psychological knowledge (e.g., expectations about others' thoughts or intentions) (Elenbaas et al., 2020). Social interactions for both youth and adults are complex and involve weighing multiple considerations, particularly in intergroup contexts. Discovering how youth think about situations in which inequalities between students are created in the classroom can inform how educators address group disparities and rectify classroom strategies that may contribute to inequalities in students' school experiences based on social identities. Recent SRD literature has examined children's and adolescents' perceptions of ethnic and gender biases about teacher allocation of leadership duties (Killen et al., 2024) and unfair treatment in the classroom setting in STEM contexts (Mulvey et al., 2024). This research has demonstrated that in these contexts, adolescents, but not children, recognize teacher biases as wrong and desire to rectify them.

What has not been investigated is youth's expectations of biased behavior regarding racial inequalities in academic recognition from a social‐cognitive developmental perspective. Understanding more about how youth perceive the likelihood of racial bias in the classroom reveals the way they attribute the intentions of teachers and peers, which may affect how youth either accept or opt to challenge racial bias in their daily lives (Rattan & Dweck, 2010). While prior research has investigated children's beliefs about prejudice malleability in a general sense (Pauker et al., 2022; Tai & Pauker, 2021), this study examines participants' expectations and reasoning about the future behavior of individuals who have displayed a racial preference in a setting salient to children's daily lives: the classroom. Our methodology uses hypothetical vignettes to investigate how children and adolescents perceive and predict others' biased behaviors. In this approach, participants' expectations about the hypothetical behavior of characters reflect their own social attitudes such as the norms they may perceive in their own environments (Crystal et al., 2008; Heck et al., 2022; Smetana & Yoo, 2022), which has important implications for how youth themselves might behave, including whether they would demonstrate the same biases (Pauker et al., 2022) or intervene if they witnessed biased behavior (Rattan & Dweck, 2010).

### Expectations about fairness and bias in the school context

School may be the first environment in which youth recognize and experience unfair treatment by authority (Okonofua et al., 2016). By age 11, children vary in their perceptions of teacher fairness, which has important consequences for their own development of moral judgment and self‐control over time (Nivette et al., 2022). Latino and White American 5–11‐year‐olds recognized discrimination as a potential reason for a teacher repeatedly rewarding same‐race students (Brown, 2006). Children also expect peers to have group norms that favor their racial or ethnic ingroup (Nesdale & Lawson, 2011; Tropp et al., 2014).

These perceptions of fairness and unfairness in their school environment inform young people's sense of social norms regarding how different groups of people are treated, which are then reflected in their expectations of others' behavior going forward. One's expectations about how another person will behave represent one's perception of the social norm in a given environment (Cooley et al., 2019; Shutts et al., 2013), such as school. Prior literature both in classic social psychology (e.g., Asch, 1958; Cialdini et al., 1990) and intergroup social developmental research (e.g., Kollerová et al., 2018; Nesdale et al., 2005; Tropp et al., 2016) has demonstrated that individuals' social attitudes and perceived social norms are often associated with their own behavior. If youth perceive teacher and peer bias in their own school environments as prior literature suggests, they may also demonstrate this expectation in the context of a hypothetical vignette. In this study, we might expect that when youth view a fictional teacher or a peer display a racial preference in academic recognition, they will expect the character to display that same preference again.

These expectations may differ between childhood and adolescence. Adolescents are often more aware of the presence of discrimination than are children (Brown & Bigler, 2005). Among early adolescents, British grade eight students expected their teachers to show gender bias (Ouazad & Page, 2013). Adolescence is also a time during which many youth strengthen their belief in the fixed nature of personality characteristics (Yeager et al., 2013), which may also apply to beliefs about bias, though little research has investigated this question. Given this evidence, the period of development between middle childhood and early adolescence may show a shift in youth's beliefs about group‐based bias, but this has not been investigated in terms of their expectations of whether bias is likely to persist at school. We may expect adolescents' expectations to reflect a belief that bias is difficult to change, while children may demonstrate an expectation that bias is more easily reduced.

While there is some history of anti‐bias education in schools in the United States (Brown, 2011), no research that we know of has investigated adolescents' expectations about whether school‐based messages aiming to promote fairness and reduce bias may change the biased behavior of teachers and peers. We therefore sought to investigate whether youth have different expectations about a biased individual's behavior in the immediate future, compared with after the individual in the scenario has received a message promoting fairness. It may be that youth's expectations reflect a belief that prejudice can change only when something has occurred to address the teacher or peer's bias.

### Expectations of teachers compared with peers

Prior research has demonstrated that children and adolescents often hold different expectations of authority figures, such as teachers, than they do of their peers. A study of Danish majority children aged 8–12 years found that children evaluated teacher exclusion of a student as less acceptable than peer‐to‐peer exclusion (Møller & Tenenbaum, 2011). A survey of American White, Black, Latine, and Multiracial high school students found adolescents were more likely to attribute teacher unfair treatment of students in a STEM class to prejudice than they were to attribute a peer's similar behavior to prejudice (Mulvey et al., 2024). Perceptions of a teacher's bias may also show age‐related change; a recent study of White, Multiracial, Black, Latine, Asian, Pacific Islander, and other ethnic‐racial background American 8–14‐year‐olds demonstrated that adolescents negatively evaluated teacher ethnic‐racial bias in allocation of classroom leadership duties, whereas children did not (Killen et al., 2024). Further, Bañales et al. (2020) demonstrated that from grade 10 to 12, Black American adolescents were increasingly likely to use structural attributions, such as systemic racism and discrimination, to explain racial achievement gaps.

Research has also demonstrated that U.S. adults believe adolescents and children's traits, such as personality and intelligence, to be more malleable than adults' (Neel & Lassetter, 2015). Chaney and Chasteen (2024) found that these assumptions also apply to beliefs about prejudice, showing that U.S. adults were less likely to confront anti‐Black prejudice the older the prejudiced individual was, in part due to the belief that the individual would not change. Thus, the same pattern may emerge in youth's expectations about classroom bias, such that youth may expect a teacher to maintain a racial preference more than they expect a peer to do so.

### Perceptions of bias favoring different racial groups

Developmental research on children's and adolescents' awareness of social inequalities in a variety of contexts has demonstrated that by middle childhood, youth are aware not only of racial categories, but also of racial inequality in which some groups are systematically advantaged (Hughes et al., 2023; Perry et al., 2025). A study that showed Black and White American children a race‐based inequality in medical resources found that 10–11‐year‐olds were both more aware of race‐based wealth disparities and more likely to rectify the inequality when Black children were disadvantaged, compared with when White children were disadvantaged (Elenbaas & Killen, 2016). In the classroom setting, U.S. adolescents ages 12–14 evaluated a teacher's denial of leadership opportunities to Latine students as less acceptable than if the teacher were to deny opportunities to White students, though the same difference in evaluations was not present among children ages 8–11 (Killen et al., 2024). Research by Olson and colleagues (2011) showed that by 7–11‐years old, U.S. youth (race and ethnicity not specified), rectified inequalities that disadvantaged Black compared with White children, but did not rectify inequalities that disadvantaged Asian compared with White children. Asian youth face their own complex set of stereotypes, at risk of peer discrimination (Rivas‐Drake et al., 2008) yet often “positively” stereotyped by teachers with high expectations of academic performance (Shi & Zhu, 2023). Still, this prior research illustrates that youth may be particularly aware of the disadvantages faced by Black students and therefore may be more likely to expect a teacher or peer to maintain their bias when it disadvantages Black students compared to when it disadvantages White or Asian students.

### Perception of racial representation at school

Another important contributor to youth's expectations about whether a teacher or peer may maintain or change in their racial preference is one's own experience of being in the racial minority or majority at school. Both Black (Johnson et al., 2024; Rivas‐Drake et al., 2014; Sellers et al., 2006) and Asian U.S. youth (Kim et al., 2023; Rivas‐Drake et al., 2008) experience discrimination at school. Experiences of being in the ethnic‐racial minority or of explicit discrimination by peers or adults have been shown to affect U.S. immigrant adolescents' perceptions of teacher fairness and school discipline (Peguero & Bondy, 2015). In the peer context, a study of fourth‐, seventh‐, and tenth‐grade U.S. White, Black, Asian, Hispanic, and other‐race youth found those with more interracial contact viewed social exclusion among peers as more wrong yet also expected race‐based exclusion to be less likely (Crystal et al., 2008). Higher levels of interracial contact may be more likely for children and adolescents who are in the racial minority in their school environment, though little research has tested the relation between this experience and beliefs about bias and its malleability in the academic context. It may be that youth who have experienced being an ethnic‐racial minority at school expect an individual's bias to persist, given their experiences with unfair treatment (Johnson et al., 2024), but it could also be the case that, consistent with findings in the peer context (Crystal et al., 2008), these youth are in fact more optimistic about inclusive behavior by teachers and peers.

### The present study

The present study investigated adolescents' and children's expectations of and reasoning about both teacher‐ and peer‐biased behavior. All participants observed two vignettes, in which (1) a teacher and (2) a student displayed a racially preferential allocation of academic recognition (only displayed the classwork of one racial group of students). Participants were assessed about their expectations and reasoning about the biased allocators' future behavior. We focused on a comparison between early adolescence (ages 12–14) and middle childhood (ages 8–11) given prior literature showing there may be an age‐related shift in awareness of group‐based bias during this developmental period (Elenbaas et al., 2016; Hughes et al., 2023; Killen et al., 2024).

### Hypotheses

Based on literature and theory reviewed above, regarding youth's awareness of bias (Brown, 2006; Nesdale & Lawson, 2011; Ouazad & Page, 2013), we predicted (H1a) that participants would expect the allocator (teacher or peer) who has shown a racial preference for academic recognition to maintain the preference by selecting a member of that racial group again. We hypothesized that this expectation would be stronger for (H1b) adolescents than for children (Brown & Bigler, 2005; Yeager et al., 2013), for (H1c) participants viewing the allocator prefer White or Asian students, rather than Black students (Killen et al., 2024), and for (H1d) participants who more strongly reported being in the racial‐ethnic minority at school (Johnson et al., 2024). We also examined participants' reasoning regarding their expectations. For concision of this manuscript, these hypotheses and results can be found in Supplemental Materials.

We predicted (H2) that participants would expect a teacher to display the preference more than they would expect a peer to do so, given prior research and theory regarding differences in individuals' expectations about behavior and attitude change in adults, compared with youth (Chaney & Chasteen, 2024; Neel & Lassetter, 2015).

Lastly, we predicted (H3) that after participants saw the allocator receive a school‐based message promoting fairness, they would be more likely to expect the allocator to give recognition equally or rectify the allocation of recognition than to maintain the biased allocation. This hypothesis was rooted in theory that change in behavior or bias occurs through learning and engagement in social interactions (Rattan & Georgeac, 2017) as well as in literature demonstrating youth's preferences for equality between social groups (Elenbaas et al., 2016).

Prior research has demonstrated mixed findings regarding effects of participant race on social cognitive outcomes in interracial inequality contexts for children and early adolescents. White American 9‐14‐year‐old children have been found to predict cross‐race social inclusion to be less likely than their Black counterparts (Cooley et al., 2019), and Black 6‐12‐year‐old American children disapproved more of interracial exclusion than their White counterparts (Luken Raz et al., 2025). However, other literature has found that Black and White American 8‐14‐year‐olds did not differ by race in evaluations of a teacher's biased allocation of leadership duties (Killen et al., 2024), as well as no differences between Black and White American 10‐ and 11‐year‐olds' decisions to rectify a racial resource inequality (Elenbaas et al., 2016). Mulvey et al. (2024) also found no racial group differences between American Black, White, Latine, and multiracial adolescents' evaluations of peer and teacher unfair behavior. Given this background and the lack of studies including Asian American as a target racial group for analysis, yet given the relevance of race to our study, we examined participant race on an exploratory basis but did not test confirmatory hypotheses about participant race.

## METHODS

### Design

This study utilized a 3 (between‐subjects condition: preferential treatment favoring Asian, Black, or White students) × 2 (within‐subjects: teacher, peer allocator) × 2 (age group: adolescents, children) design. Participants were randomly assigned to one of three between‐subjects conditions to see a vignette‐based survey in which the allocating characters show a preference for either Asian, Black, or White students. All participants viewed two vignettes representing the within‐subjects conditions, one in which a teacher allocated academic recognition and one in which a peer was the allocator. Participants viewed the two vignettes in a randomized order.

### Participants and procedure

Participants included *N* = 303 (154 girl, 145 boy, 4 other, 47.5% Black/African American, 36.9% White/European American, 15.5% Asian American/Pacific Islander) U.S. children (*n =* 172, ages 8–11, *M*_age_ = 10.34) and adolescents (*n =* 131, age 12–14, *M*_age_ = 13.09) from a major metropolitan area in the Mid‐Atlantic United States. Participants were from middle income to high‐middle income, given that the median household income in the area is $126,244 (Census Reporter, n.d.). We recruited participants from local public and private schools, after‐school programs, and summer camps in 2023 and 2024. Youth between ages 8 and 14 were invited to participate regardless of racial background. For analyses presented in the current study, we retained only participants who self‐identified as Black, White, or Asian due to these being the three racial groups depicted in the stimuli. An a priori power analysis in the program G*Power (Faul et al., 2009) determined that a sample size of 252 would allow us to test our hypotheses with an effect size of .20. To account for incomplete responses and achieve a sample in which we could analyze each racial group, we collected a total of 303 participants. Tables S1 and S2 provide further information on participant demographics.

The study was approved by the Institutional Review Board at the University of Maryland, College Park. We obtained parental consent and child assent for all participants. Among the 353 parents who completed consent forms, there was an 86% consent rate, for a total sample of 303 participants. Youth who received a consent form received a keychain. All participants completed the survey individually, in their site environment (e.g., classrooms, school cafeterias, or summer camps), supervised by a researcher to whom participants could ask questions. Most participants (*n* = 233) completed the survey on Qualtrics, though at sites without access to laptops or internet connection, participants (*n* = 70) filled out the survey in hard copy. Researchers gave the same instructions to all participants, and no differences were detected for responses from those who completed the survey online versus in hard copy. Participants took between 20 and 30 min to complete the survey in both formats.

### Vignettes and measures

This study utilized a vignette methodology, reflective of an extensive body of research in social‐cognitive development (Heck et al., 2022; Smetana & Yoo, 2022). From this approach, everyday interactions are described accompanied by illustrated pictures (see Burkholder et al., 2021; Killen et al., 2024). This is done to enable both children and adolescents to follow the set‐up and understand the nature of the questions posed. In this study, participants read two vignettes in which students of only one racial group received recognition by having their academic work displayed on the classroom wall. The two vignettes varied by the role of the character (teacher or peer) who made the racially biased choice in recognizing student work, such that in one vignette, a teacher made the racially biased selection of recognition of students' schoolwork, and in the other vignette, a peer “student helper” made the selection. The events of the vignettes were developed based on research demonstrating the presence of racial bias in classrooms (İnan‐Kaya & Rubie‐Davies, 2021; Peterson et al., 2016; Staats, 2016) and youth's awareness of race and racial inequalities in the school context (Hughes et al., 2023; Killen et al., 2024; Mulvey et al., 2024).

**FIGURE 1:**
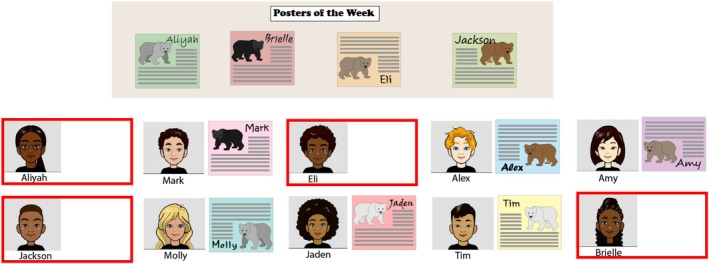
Example stimuli of racial preference in academic recognition, black preferred condition.

Stimuli depicted the race of characters using skin color, facial features, and hair type, reflecting the historical social construction of racial categories using these perceived physical differences (Perry et al., 2025); see Figure 1. We use the term race to describe the stimuli here, as participants only received this visual indication of group differences. References to ethnicity can imply a shared ancestry, traditions, language, and other generationally passed‐down practices, which were not depicted in the stimuli (Nagel, 1994; Perry et al., 2025). All characters were depicted with the same facial expressions and clothing, and an equal number of boys and girls were chosen in the initial display of the bias. Pilot testing confirmed that youth in the sample age range distinguished between the racial groups depicted in the stimuli and understood the events of the vignettes as well as the assessments. The full protocol can be found in the Supplemental Materials.

The **teacher vignette** introduced Ms. Parker as the allocating character, reading, “These are some students in Ms. Parker's class. The students in Ms. Parker's class studied different types of bears. They made posters about the bears they learned about. Here are some of the students and their posters. Everyone did a really good job! The students really like it when their poster is put up on the wall. Everyone hopes their poster will be chosen. Ms. Parker will choose 4 posters that will hang on the classroom wall. These are the students whose posters Ms. Parker picks to go on the wall.” The topics of the posters in the vignettes were varied each time participants saw students make new posters (bears, planets, birds, and plants) to keep the stories and stimuli engaging and distinct. Other than the poster topic and the initial setup of the teacher and peer allocators, the text of the two vignettes was identical. We did not provide any visual or descriptive information about the race of the teacher or peer allocator.

The **peer vignette** introduced Sam as the allocating character with equivalent text explaining the posters and presenting a different set of students. The vignette explained, “Ms. Sanders is not at school today. Sam is a student helper for the substitute teacher. Sam is in charge of choosing 4 posters that will hang on the classroom wall. These are the students whose posters Sam picks to go on the wall.” In each vignette, this introduction was followed by stimuli depicting the allocator's selection of four students, all from one racial group (Asian, Black, or White, based on between‐subjects condition). See Figure 1.

**FIGURE 2:**
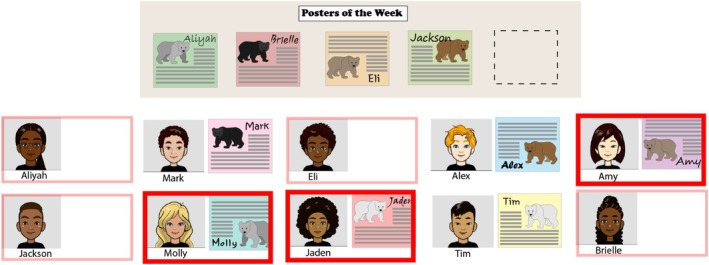
Example stimuli for expectation of allocator bias.

After viewing the teacher or peer allocator show the initial racial preference, participants read, “Later, Ms. Parker/Sam notices that there is room for one more poster on the classroom wall. Ms. Parker/Sam wants to hang one more poster, but can't decide between [Molly, Jaden, and Amy's] posters.” The three students presented as options represent each of the three racial groups: Asian, Black, and White. The three students were always the same gender. See Figure 2.

#### Expectation of bias

Participants were then asked, “Who do you think Ms. Parker/Sam will pick to hang their poster on the last spot on the wall?” Participants selected one of the three students.

#### Fairness message

Participants then read that the allocator received a school‐based message promoting fairness at a meeting for teachers (teacher vignette) or student assembly (peer vignette). In each vignette, the message communicated that people may “assume students who look different don't know as much. But it's important to give everyone the same opportunities.” Both the teacher and peer were said to have enjoyed their meeting and assembly, respectively. Following the fairness message, participants responded to two manipulation check items, “Who attended the meeting/assembly?” and “What did they learn at the meeting/assembly?” All participants passed the manipulation checks within two attempts.

#### Expected likelihood of biased, equal, or rectifying allocations

Participants then read that the day after the meeting/assembly, students made new posters the next day and were reminded of how the teacher/peer allocated recognition the last time. They read that the allocator would now choose three students' posters to display on the wall. The vignettes then presented three potential allocations of recognition the allocator could make to recognize the current set of student work: (1) *Biased allocation* (three students from the same racial group as the first time), (2) *Equal allocation* (one student from each racial group), and (3) *Rectifying allocation* (only students from the racial groups not chosen the first time). For each potential allocation, participants were asked, “What if Ms. Parker/Sam picked *these* students' posters this time? How **likely** is Ms. Parker/Sam to pick ***these*** students' posters?” Participants responded on a Likert‐type scale, from 1 = *Really not likely* to 6 = *Really likely*.

#### Perceived numeric racial representation

After both vignettes, participants responded to three items assessing their perception of racial representation in their own school environment, adapted from prior literature (Crystal et al., 2008; Kaufman & Killen, 2026). Items read, “Think about your [classes at school/grade at school/whole school]. How many kids in your [class(es)/grade/school] are the same race or ethnicity as you?” Participants responded to each item on a Likert‐type scale, from 1 = *None* to *5 = All*. A mean composite was used for perceived numeric racial representation (PNRR) (Cronbach's α = .86). A PNRR score of 5 indicated a participant perceived high representation of their own racial‐ethnic group at their school, and a score of 1 indicated a participant perceived themself to be in a racial‐ethnic minority at their school. See Table S1 in Supplemental Materials for analyses of PNRR and participant race.

### Analytic approach

We first tested mixed effects models with participants clustered into recruitment sites. Given large variability in site size and low ICC values (ICCs < .02), we proceeded with single‐level models and mixed effects models within participants for testing hypotheses about repeated measures. These confirmatory analyses were conducted using R (R Core Team, 2023) and were preregistered along with our hypotheses and analytic code on Open Science Framework. All analyses tested the effects of age group, condition, participant race, and PNRR. We conducted Holm‐Bonferroni‐adjusted pairwise comparisons to follow up findings of significant group differences. We also tested the effect of vignette order (teacher allocator or peer allocator first) in every model and found no significant effects of order.

To test our H1, we conducted multinomial logistic regressions regressing expectations (forced‐choice selection of an Asian, Black, or White student) on condition, age group, PNRR, and participant race. We examined the effect of condition for the respective outcome category to assess if participants' expectations of bias differed by which racial group they saw preferred. We conducted these models separately for the teacher and peer allocator vignettes.

To test our H2, we first created a dichotomized version of the initial expectation of bias measure, such that 1 = the participant expected the allocator to maintain the preference (choose another student of the same race), and 0 = the participant expected the allocator to choose a student from another racial group. Using a generalized linear mixed effects model with a logit link, we regressed this binary outcome onto the following predictors: allocator (teacher/peer), age group, condition, participant race, and PNRR, with a random intercept for participant, to test for the allocator repeated measure and fixed effects for predictors. We also conducted linear mixed effects models using this same set of predictors on participant likelihood expectations of the biased allocation after the fairness message.

To test our H3, we conducted mixed effects models with a random intercept for participants and fixed effects for predictors: age group, allocation type (biased, equal, rectifying), condition, participant race, and PNRR. Models were conducted separately for each vignette. For each model, we tested for interactions with and main effects of participant race. When the interaction term was not significant, we removed the interaction term to prefer the simpler model but included participant race in every final model.

Our analytic approach, coded response categories, and results regarding reasoning can be found in Supplemental Materials (Table S3).

## RESULTS

### Expectations of teacher and peer racial preference

**FIGURE 3:**
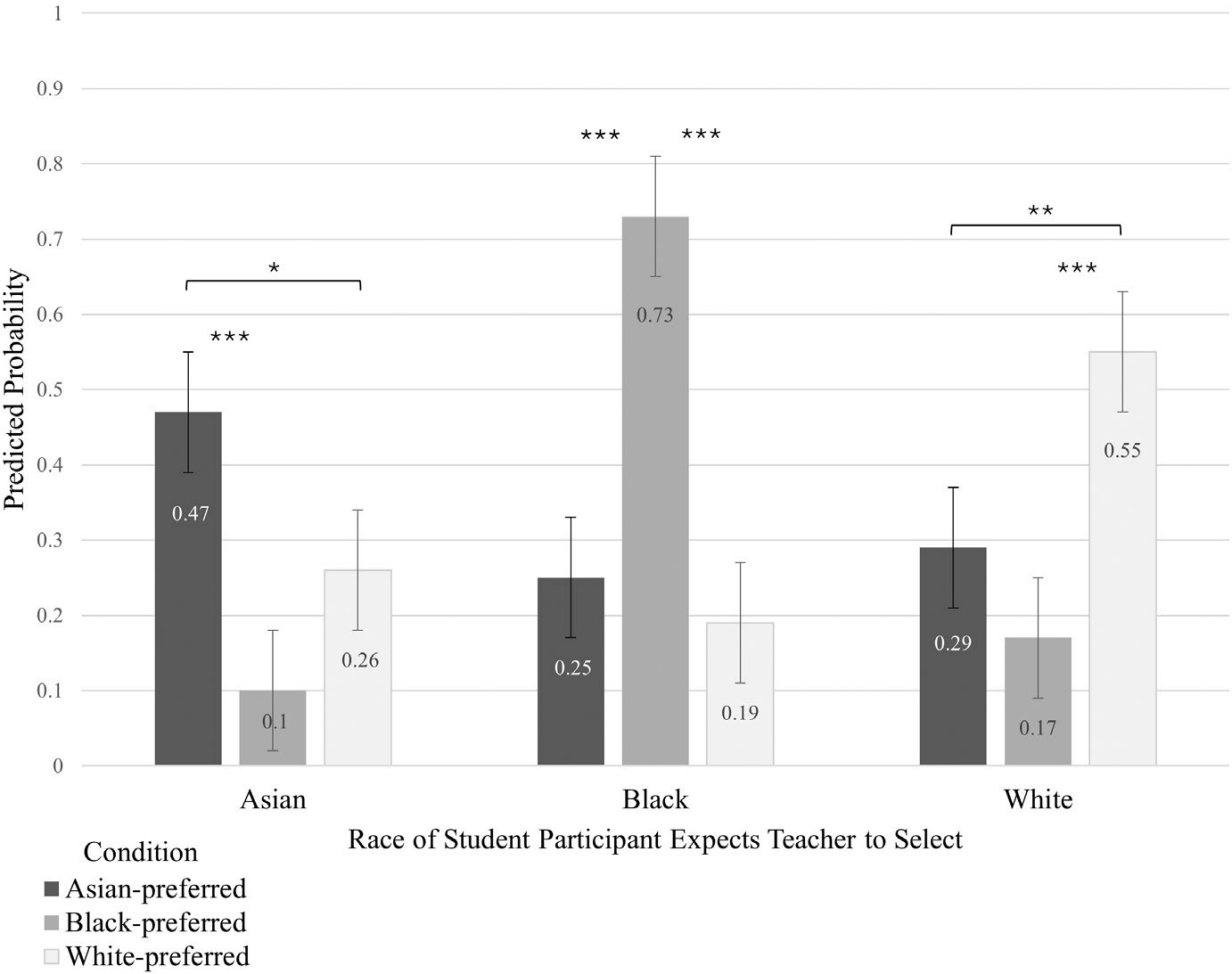
Participant expectations of initial teacher bias in academic recognition, by condition.

**FIGURE 4:**
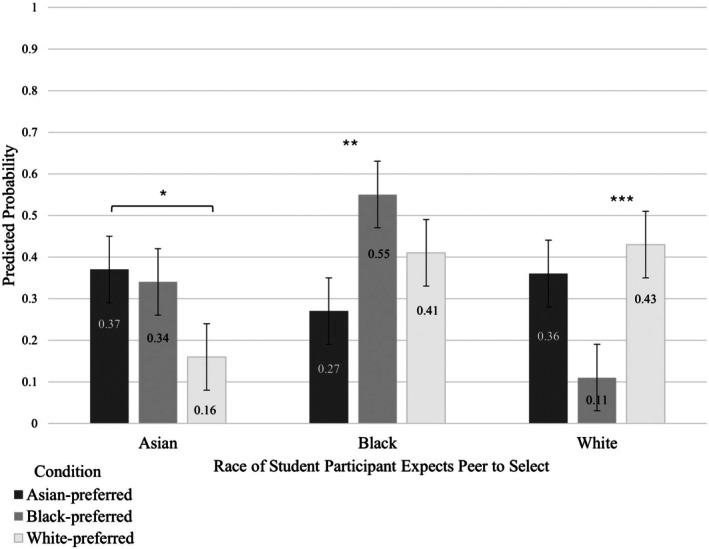
Participant expectations of initial peer bias in academic recognition, by condition.

In multinomial logistic regressions testing our H1, we found significant effects of condition for both vignettes, confirming our hypothesis that participants would expect both the peer and teacher allocators to continue their preference for the same racial group, before seeing the fairness message. Full odds ratios can be found in Supplemental Materials, and predicted probabilities are displayed in Figures 3 and 4. We conducted follow‐up Holm‐Bonferroni‐adjusted pairwise contrasts by condition on predicted probabilities (Table 1), which confirmed the finding, also revealing that this pattern was more strongly and consistently demonstrated in the teacher vignette (Figure 3).

**TABLE 1:** Predicted probability of expecting selection of previously preferred racial group by vignette.

Condition	Predicted probability (SE)	*p* (vs. Asian‐preferred)	*p* (vs. black‐preferred)	*p* (vs. white‐preferred)
*Teacher vignette*				
Asian‐preferred	.47 (.05)	–	<.001	.016
Black‐preferred	.73 (.05)	<.001	–	<.001
White‐preferred	.55 (.05)	.004	<.001	–
*Peer vignette*				
Asian‐preferred	.37 (.05)	–	.69	.013
Black‐preferred	.55 (.05)	.002	–	.107
White‐preferred	.43 (.05)	.300	<.001	–

*Note*: Predicted probabilities were estimated from multinomial logistic regression models. Chance‐level probability is .33. Significance levels reflect Holm‐Bonferroni adjustments of pairwise contrasts on predicted probabilities. Full multinomial odds ratios are reported in supplemental materials. *N* = 303.

In the teacher vignette, participants consistently expected the teacher to maintain the racial preference, regardless of which racial group participants saw the teacher prefer. In the peer vignette, participant expectations were more moderate and varied by condition (Figure 4). Specifically, participants in the Asian‐preferred condition were more likely to predict a White student would be chosen than those in the Black‐preferred condition (*p* = .001). In the Black‐preferred condition, there was the clearest pattern of expectations of the peer maintaining their racial preference, while in the Asian‐preferred condition, participants did not as strongly expect the peer to maintain the racial preference. Black participants were also more likely than White participants to expect a Black student to be chosen by a peer, regardless of condition and age (*OR:* 2.00, 95% CI [1.08, 3.71], *p* = .028). White participants were less likely than Asian participants to expect the peer to pick a White student (*OR:* .50, 95% CI [.27, .93], *p* = .028).

#### Age group differences

**FIGURE 5:**
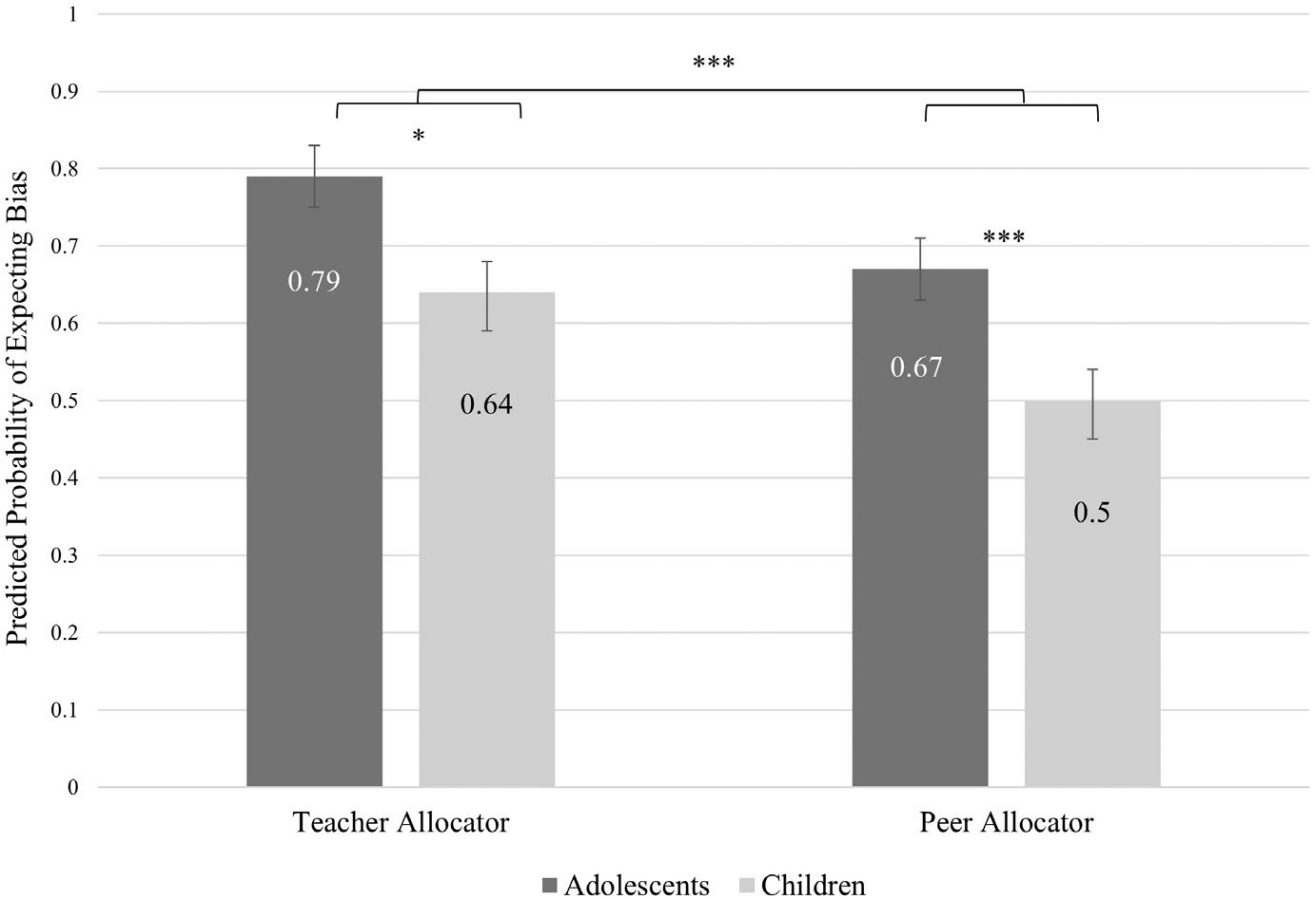
Predicted probability of expecting racial bias from a teacher or peer allocator.

In separate logistic regressions to probe the effect of age group in each vignette, we confirmed our hypothesis (H1b) that adolescents were more likely than children to expect the peer to show the preference (*OR*: 2.37, 95% CI [1.45, 3.91], *p* < .001), as well as the teacher (*OR*: 1.82, 95% CI [1.11, 3.02], *p* = .019). See Figure 5.

#### Differences by preferred racial group

As expected, youth who saw the teacher and peer prefer White or Asian students were more likely than those who saw Black students to expect the teacher to rectify their initial bias (by selecting a student of another race). In the peer vignette, participants in the Black‐preferred condition were more likely than those in the White‐preferred condition (*OR*: 1.8, 95% CI [1.01, 3.24], *p* = .046) as well as those in the Asian‐preferred condition (*OR*: 2.21, 95% CI [1.24, 3.99], *p* = .008) to expect the peer to maintain the preference, confirming our H1c. This was also true in the teacher vignette, in which participants in the Black‐preferred condition showed higher log odds than those in the White‐preferred condition (*OR*: 2.21, 95% CI [1.22, 4.03], *p* = .009) and Asian‐preferred condition (*OR*: 2.97, 95% CI [1.66, 5.41], *p* < .001) to expect the teacher to maintain the preference. Contrary to our H1d, we found no effect of PNRR on participants' initial expectation of bias.

### Expectations about teacher versus peer racial preference

Participants were more likely to predict the teacher would select another student of the same racial group than that the peer would (*OR:* 1.78, 95% CI [1.27, 2.50], *p* < .001), confirming our H2 (Figure 5). This was consistent with findings from H1, where we saw a clear pattern of expectations of maintained racial preference in the teacher vignette, whereas in the peer vignette participants' expectations were less consistent and varied by condition. We did not find this difference after the fairness message; there was not a significant difference in participants' likelihood expectations of the teacher making another racially biased allocation of recognition compared with the peer.

### Expectations of racial preference after a fairness message

Overall, youth expected both a teacher and peer to maintain their racial preference in academic recognition, even after the allocator received a prompt to treat all students fairly. This finding was contrary to our H3. Specifically, in the peer vignette, participants rated the biased allocation as *more* likely than both the equal (*β* = .40, 95% CI [.26, .52], *p* < .001) and rectifying (*β* = .51, 95% CI [.37, .64], *p* < .001) allocations. In the teacher vignette, participants rated the biased allocation as more likely than both the equal (*β* = .16, 95% CI [.02, .30], *p* = .023) allocation and the rectifying allocation (*β* = .32, 95% CI [.18, .46], *p* < .001) and rated the rectifying allocation as significantly less likely than the equal allocation (*β* = − .16, 95% CI [−.30, −.02], *p* = .029). We found no significant effects of condition or age on participants' likelihood ratings of teacher and peer allocations after the fairness message.

#### Differences by perceived numeric racial representation

When testing our expectation that *lower* PNRR would be associated with a *higher* expected likelihood of the teacher and peer showing the racial bias, we found the opposite effect in measures after the fairness message. Rather, the more strongly youth reported feeling part of the racial‐ethnic *minority* at school (low PNRR), the *less* likely they expected the repeated bias to be in both the peer (β = .18, 95% CI [.06, .29], *p* = .003) and teacher (β = .13, 95% CI [.02, .25], *p* = .027) vignettes.

## DISCUSSION

The present study is the first to investigate youth's expectations about whether teacher and peer racial preferences can change in the classroom context. Understanding whether youth expect a bias to be maintained from one day to the next has important implications for their behavior in this setting, in which it is common for racial biases to be displayed by teachers and peers (Cooley et al., 2016; Peterson et al., 2016; Staats, 2016).

### Expectations about initial display of a racial bias

Participants expected both teachers and peers to maintain their racial preferences, as we hypothesized. Expectations of a repeated racial preference indicate youth's awareness of the initial preference, which reflects research demonstrating that adolescents and children recognize the presence of teacher bias based on social groups such as race, ethnicity, or gender (Brown, 2006; Nivette et al., 2022) as well as prior findings showing that children and adolescents are aware of peer ingroup preferences based on their racial or ethnic group (Graham et al., 2011).

Interestingly, participants who saw Black students preferred were most likely to expect the racially preferential treatment by the peer to continue, compared with participants who saw Asian or White students preferred. Prior literature has illustrated that among Black and White American children, White children were perceived as more socially inclusive of Black peers than vice versa (Tropp et al., 2014), and Black American children have been found to form strong same‐race friendships when in White‐majority school environments (Wilson & Rodkin, 2011). It could be that participants have perceived these patterns among their own peer groups and therefore were more likely to assume that, though the vignette did not depict the race of the peer allocator, a preference for Black students represented this type of same‐race friendship preference. Though this cannot be determined from the data collected here, future studies could examine this possibility through collecting further reasoning responses or measures asking participants to guess the racial group of the peer allocator character.

We also found that participants who saw the peer prefer Asian students were least likely of all conditions to expect the peer to maintain the racial preference. In the United States, Asian students face peer discrimination (Kim et al., 2023; Kogachi & Graham, 2021), often experiencing both the effects of being in a racial‐ethnic minority at school, as well as the deleterious effects of the model minority stereotype. It could be that youth in this study had some awareness of the peer discrimination faced by Asian students and therefore were less likely to expect that racial preference to continue. However, more research is needed examining Asian students as a target racial group as well as a participant group to gain a better understanding of children and adolescents' awareness of the bias faced by this peer group.

### Expectations about the teacher compared with the peer

As we had predicted, we found that youth expected the teacher to be more likely than the peer to maintain a racial group preference in academic recognition. This is consistent with prior findings in a STEM classroom context, in which adolescents were more likely to assume a teacher's unfair treatment of a student was due to prejudice than to assume the same cause of a peer's similar behavior (Mulvey et al., 2024).

This finding adds to prior literature demonstrating that individuals tend to have more incremental (growth) mindsets regarding traits of younger people, thus expecting their personalities to be more malleable or likely to change, while showing more entity mindsets (fixed) regarding older individuals (Neel & Lassetter, 2015). Research specifically examining prejudice malleability mindsets has shown this same effect regarding target age (Chaney & Chasteen, 2024). This study provides preliminary evidence that in a classroom context adolescents are more likely to expect bias to continue than are children. After the fairness message, we did not find a difference in expectations of the teacher making another racially biased allocation of recognition, compared with the peer. Thus, while youth did not expect the teacher or peer to be fairer after the message, the fairness message somehow “washed out” differential expectations about teacher and peer bias. Additional research is needed to further examine youth's perceptions of the efficacy of school‐based messaging promoting fairness and inclusion to change adults versus students' behavior.

### Adolescents' versus children's expectations of bias

In their initial predictions of how the teacher and peer would next allocate academic recognition after showing a racial group bias, adolescents expected both the teacher and peer allocator to be more likely to continue the racial preference than did children. These findings are consistent with prior literature that awareness of teacher bias and unfair treatment based on group identity is higher among adolescents than among children (Brown & Bigler, 2005; Killen et al., 2024). Adolescents are more likely than children to show racial or ethnic ingroup preferences when it comes to peer group affiliations, and same‐race peer affiliations strengthen with age across early adolescence (Kogachi & Graham, 2021). Thus, adolescents may then expect that peer character is also likely to make an allocation of classroom recognition based on a racial group preference. This study adds to this prior work by demonstrating awareness using the expectation measure as well as by demonstrating this age‐related difference in awareness of racial bias at school in the context of academic recognition. Further, prior research shows that during adolescence, it is common for youth to adopt more fixed mindsets about other personal characteristics (Yeager et al., 2013). This study presents novel evidence to this effect regarding mindset about prejudice specifically in the age range from middle childhood to early adolescence, though future research is needed to understand more about the circumstances in which youth expect prejudice to be malleable.

Though stage‐based theory of cognitive development may suggest that age‐related differences in responses in a vignette‐based task reflect advances in hypothetical reasoning gained in adolescence (Piaget & Inhelder, 1969), research has demonstrated that children are in fact very capable of hypothetical reasoning about vignettes across a variety of contexts. For example, theory of mind research has demonstrated that school‐aged children can perceive others' biases, preferences, and intended behaviors (Hughes & Leekam, 2004). Further, significant research on developmental social cognition has used vignettes to examine children's hypothetical reasoning regarding group‐based social hierarchies, including race, from early childhood through adolescence (for a review, see Heck et al., 2022). In a study in which 6–12‐year‐olds made predictions about who would occupy different occupations, children's hypothetical judgments indicated an understanding of racial segregation in the job force, even among 6‐year‐olds (Bigler et al., 2003). This study and many others (e.g., Essler & Paulus, 2021; Olson et al., 2011; Yang & Dunham, 2022) demonstrate not only that children are capable of engaging in reasoning and making judgments about hypothetical events depicted in vignettes, but that their judgments reflect their own inferential social cognition based on their observations, experiences, interpretations, and evaluations of their everyday social world, rather than merely developmental advances in hypothetical reasoning as a cognitive capacity. Given this body of evidence, we interpret our finding that adolescents expected the teacher and peer to initially demonstrate the racial bias, more so than did children, to represent age‐related change in awareness of racial bias and, therefore, cannot be explained solely by maturation in metacognition or hypothetical reasoning ability. However, this does not explain our unexpected finding that there were *no* differences between age groups in their expectations about the most likely allocation of recognition after the fairness message. Rather, the majority of both children and adolescents expected the teacher and peer characters to maintain a biased allocation after the message. Future research should examine perceptions of different fairness messages from middle childhood through adolescence to further probe what may have driven this finding.

### Expectations after the fairness message

Unexpectedly, we found that participants thought the biased allocation was more likely than the equal or rectifying allocations of academic recognition after both the teacher and peer allocators were exposed to the message promoting fairness. We know from prior research that youth are aware of discrimination by teachers (Nivette et al., 2022; Ouazad & Page, 2013) and peers (Benner & Wang, 2017; Douglass et al., 2016) in their own school contexts. Yet, youth can perceive the prejudice that may lead to such discriminatory behavior as malleable (Pauker et al., 2022). This study tested whether a simple message promoting fairness was enough to shift youth's perception of a racial bias, such that youth would expect the teacher or peer to show the bias less after receiving the message. It appears that, understandably, youth may perceive racial prejudice at school to be more challenging to change than what can be accomplished from a single message promoting fairness. Though youth may have perceived the message as valuable, it may be that the one‐time nature of the message limited their expectations of its efficacy. Thus, this finding does not necessarily indicate that youth simply have a “fixed” mindset about prejudice, wholesale. Future research is also needed to further investigate prejudice malleability beliefs across different circumstances and contexts, such as investigating whether youth expect repeated exposure to messaging promoting equal treatment to be more effective, as even children and adolescents may intuit or have personally experienced that behavior change often requires stronger intervention. This is important work given that when individuals do view prejudice as changeable, they are more likely to confront this behavior (Chaney & Chasteen, 2024).

### Effect of perceived numeric racial representation

In another unexpected result, we found that the more participants reported experiencing school as a racial‐ethnic minority (low PNRR), the less likely they expected the biased allocation to be in both vignettes. Put another way, youth who were in a minority racial‐ethnic group in their own school environment thought both teacher and peer racial bias was more likely to change after the fairness message, regardless of participants' own racial group. It could be that this represents some element of system justification, in which even individuals who are in systemically disadvantaged social groups often support the systems which perpetuate inequality, as this set of beliefs has been demonstrated particularly among racially and ethnically marginalized youth (Biedron et al., 2024). Another explanation could be that youth who are in a racial or ethnic minority group at school by necessity have engaged in more cross‐race interactions with teachers and peers. These interactions may involve discrimination, but they may also be positive experiences which improve expectations of outgroup inclusivity (Killen et al., 2022; Tropp et al., 2014). Thus, it may be that the association between more prejudice malleability beliefs and higher interest in interracial interactions (Carr et al., 2012; Pauker et al., 2022) also functions in the other direction: youth who have had more opportunities for interracial interactions may develop a stronger expectation of prejudice malleability. Future research is needed to explore this directionality more intentionally, particularly in the school context.

### Limitations and future directions

This study presents a first step at examining children's and adolescents' expectations about racial prejudice when displayed by a teacher or peer in the classroom context. However, it was beyond the scope of the design of this study to systematically vary the degree of the racial preferential treatment displayed or the intensity of the intervening message promoting fair treatment. While in this specific scenario we found that youth did not expect a teacher or peer to engage in more equal treatment of students after the fairness message, future studies should investigate the circumstances in which youth do perceive prejudicial behavior in the classroom as likely to change, demonstrating a more incremental mindset about prejudice. Further, future research should examine whether having a generally more incremental implicit theory about personality, i.e., a stronger belief in the malleability of traits, is associated with youth's perceptions of the malleability of classroom bias, specifically.

Further, due to limitations in protocol length to avoid fatigue for our youngest participants, and to examine differences in between‐subjects conditions regarding target racial groups, the design of our study did not focus on variation of the fairness message or specifically on using equivalent repeated measures before and after the fairness message. However, additional research would be useful to more intentionally investigate identical measures of expectations before and after a fairness message, as well as whether a condition with no fairness message or conditions with different phrasing in the fairness message may have yielded different findings. This would elucidate under what circumstances youth perceive bias as potentially changeable. Further, while research on adults' more malleable mindset about prejudice has demonstrated that this set of beliefs predicts a higher likelihood of confronting prejudice (Rattan & Dweck, 2010) and other literature demonstrates that the belief prejudice can change relates to behavioral outcomes including interracial interactions (Carr et al., 2012; Pauker et al., 2022), future research should also examine whether youth's expectations about classroom bias in a hypothetical context predict their own behavioral outcomes, such as confronting biased behavior or engaging in positive interracial interactions.

Our findings contribute new knowledge about perceptions of teacher and peer fairness in the context of academic recognition of Asian, Black, or White children in the U.S. The results here indicate some differences in how youth expected preferential treatment to continue depending on which racial group was favored, yet these effects may not generalize to different racial or ethnic target groups not examined here, or to other domains of social identity. Future research is needed to further explain the mechanisms contributing to youth's differential expectations about these racial groups as well as to explore youth's expectations about classroom preferential treatment in the context of gender, socioeconomic, or other social group prejudices, which are also likely to permeate children's school experiences (Kaufman et al., 2024). It is also the case that findings from our economically advantaged sample may not generalize to all socioeconomic groups. Future research should examine children's and adolescents' perceptions of classroom racial bias across a broader range of socioeconomic backgrounds.

### Implications and conclusions

It is crucial to understand young people's expectations about social group bias and how these perceptions may change with age. Among adults, individuals who hold a more incremental theory of personality are more likely to confront prejudice (Rattan & Dweck, 2010). This study is a first step in response to literature calling for further research examining expectations about the repetition of bias in intergroup contexts (Rattan & Georgeac, 2017) and from a developmental perspective focused on adolescents (Tai & Pauker, 2021).

This study expanded on prior findings by demonstrating that by early adolescence, youth are more likely than in middle childhood to expect both a teacher and a peer to maintain a racial bias in academic recognition, but neither age group expected that a one‐time message promoting fairness would be sufficient to make this racial preference less likely in the future. In terms of implications for educators and school policymakers, this study demonstrates that students notice when bias is present in their classrooms from both teachers and peers, but that they may perceive it takes repeated and consistent intervention to change. This could support the need for consistent programming for both teachers and students that promotes the values of fairness and equality, in order for students to feel that their schools are environments in which they can expect fair treatment from all racial and ethnic backgrounds. Children's and adolescents' expectations about bias in the classroom reveal their perceived norms about prejudiced behavior in their own social world, which may give insights into how they would respond when faced with such behavior in their everyday lives.

## AUTHOR CONTRIBUTIONS

**Elise M. Kaufman:** Conceptualization; methodology; data curation; investigation; formal analysis; funding acquisition; project administration; writing – original draft. **Marley B. Forbes:** Formal analysis; writing – review and editing. **Melanie Killen:** Conceptualization; methodology; supervision; funding acquisition; resources; writing – review and editing.

## FUNDING INFORMATION

This project was supported in part by a Clara Mayo Award to Elise M. Kaufman from the *Society for the Psychological Study of Social Issues*. Melanie Killen was supported, in part, by grants from the National Science Foundation, BCS#1728918, and the Eunice Kennedy Shriver National Institute of Child Health and Human Development, R01HD093698.

## CONFLICT OF INTEREST STATEMENT

Authors have no known conflicts of interest.

## ETHICAL APPROVAL STATEMENT

This study was conducted in accordance with the ethical standards of the APA and was approved by the University of Maryland Institutional Review Board and was approved as project [1946842‐2] on September 27, 2022.

## CONSENT STATEMENT

Parental consent and child assent were obtained for all participants in accordance with IRB ethical standards.

## PREREGISTRATION STATEMENT

The hypotheses and analyses presented here were preregistered on the Open Science Framework (OSF) at the following URL: https://osf.io/ytgjd/overview. The analytic code necessary to reproduce the analyses presented here is available on OSF. The protocol and stimuli are included in the supplementary materials.

## Supporting information


**Table S1:** Mean Perceived Numeric Racial Representation (PNRR) by participant race.
**Table S2:** Frequencies of participant household income range.
**Table S3:** Conceptual categories used to code reasoning for expectation of bias.

## Data Availability

Data can be made available upon request through email to the corresponding author.
