# Supplementary material for: Adolescents' and children's expectations of fairness and bias in the classroom
Source: J Res Adolesc. 2026 Jul 19;36(3):e70236. doi: 10.1111/jora.70236 (PMC13382098; doi:10.1111/jora.70236)
Supplement: Supplementary file 1 — Table S1: Mean Perceived Numeric Racial Representation (PNRR) by participant race. Table S2: Frequencies of participant household income range. Table S3: Conceptual categories used to code reasoning for expectation of bias. [file JORA-36-0-s001.docx]

**Adolescents' and Children’s Expectations of Fairness and Bias in the Classroom**

Supplemental Materials

**Sample Descriptive Statistics**

**Table S1**

*Mean Perceived Numeric Racial Representation (PNRR) by Participant Race*

|  |  | Perceived Numeric Racial Representation  (*1 = None, 5 = All*) | | |
| --- | --- | --- | --- | --- |
|  | *n* | *M (SD)* | *Min* | *Max* |
| Asian/Asian American | 47 | 3.21 (.68) | 2.00 | 4.33 |
| Black/African American | 144 | 3.60 (.78) | 1.33 | 5.00 |
| White/European American | 112 | 3.50 (.83) | 1.33 | 5.00 |

*Note:* Participant-reported race and PNRR. A Levene’s test for equal variances showed that variance of PNRR across racial groups did not differ significantly, *F*(2, 299) = .44, *p* = .64. We also conducted a one-way ANOVA of PNRR by participant race, which indicated that Asian and Black participants differed significantly in PNRR (*p* = .02), but neither group differed significantly from White participants. Given this finding, we took the approach of analyzing each racial group separately in our planned preregistered models, including participant race and PNRR as covariates in each model. We tested the significance of interactions between these predictors on an exploratory basis but did not find any interactions to be significant. We tested for multicollinearity of PNRR and participant race in each model and did not find multicollinearity to be present (VIFs < 1.07).

*N* = 303

**Table S2**

*Frequencies of Participant Household Income Range*

| Household Income Range | *n* |
| --- | --- |
| $30,000-$60,000 | 3 |
| $60,000-$90-000 | 3 |
| $90,000-$120,000 | 7 |
| $120,000-$150,000 | 10 |
| $150,000-$180,000 | 9 |
| $180,000-$210,000 | 16 |
| > $210,000 | 103 |
| No response | 152 |

*Note:* Optionally parent reported. *N = 303*

**Protocol**

Participants were randomly assigned to one of three conditions: (1) Asian students preferred, (2) Black students preferred, or (3) White students preferred. Stimuli showed a class of ten students, five of whom were of the race preferred by the teacher. This number was determined through pilot testing and to allow the image to be fully visible at once on Chromebook screens for children completing the survey at school. Posters were normed during pilot testing to be of equal quality. Pilot testing indicated that participants in the 8-14 age range were able to distinguish the racial identities of the students depicted in the illustrations.

All participants viewed both within-subjects conditions of teacher-allocator vignette and peer-allocator vignette in randomized order. Protocol text and stimuli from the Asian-preferred condition are below, with the teacher vignette first, followed by the peer vignette. Black and White preferred conditions stimuli was equivalent, though with the respective group shown receiving the racial preferential treatment. Full stimuli can be made available from the first author upon request.

**Manipulation Check** *(Seen by all participants to ensure understanding of stimuli. All participants passed within two attempts.)***:**

Today you will read about different classes at a school. In each classroom, there is a wall where posters are put up. Here is the wall in a classroom where they put up students' posters.


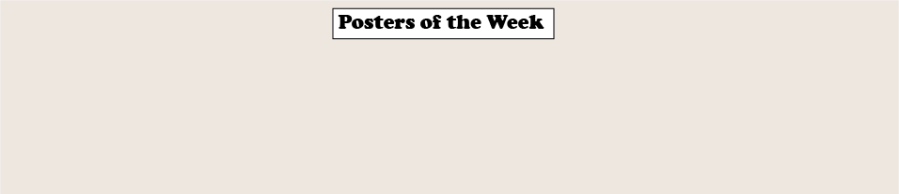


In this classroom, one student does not have their poster, because it is on the wall.


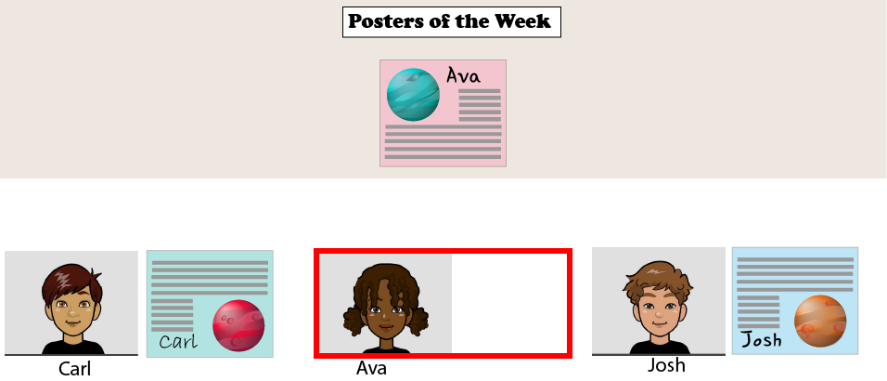


Whose poster is on the wall?

*Students click to select on Qualtrics or write in their response in hard copy.*

Great job! Now let’s move on to the stories.

**Teacher-allocator vignette:**

Today you will meet students in two classes at a school. The first one is Ms. Parker’s class. Here are some of the students in Ms. Parker’s class.

Students in Ms. Parker’s class made posters about different types of bears they learned about in class. Here are some of the students and their posters. Everyone did a really good job!

The students really like it when their poster is put up on the wall. **Everyone hopes their poster will be chosen**.

Ms. Parker will choose **4 posters** to hang on the classroom wall. These are the posters Ms. Parker picks.


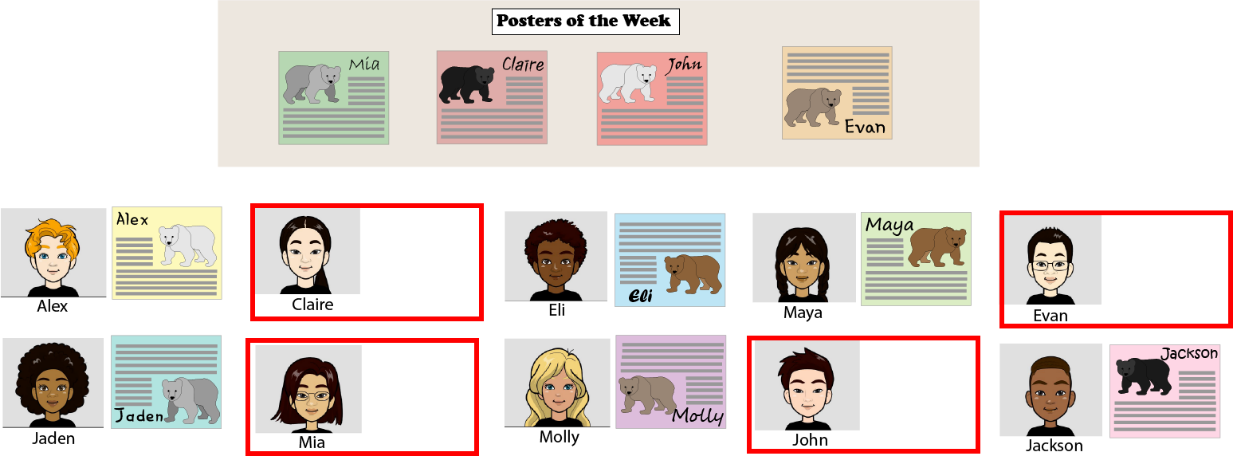


Later, Ms. Parker notices that there is room for one more poster on the classroom wall. Ms. Parker wants to hang one more poster, but can’t decide between **Molly, Jaden**, and **Maya**’s posters.


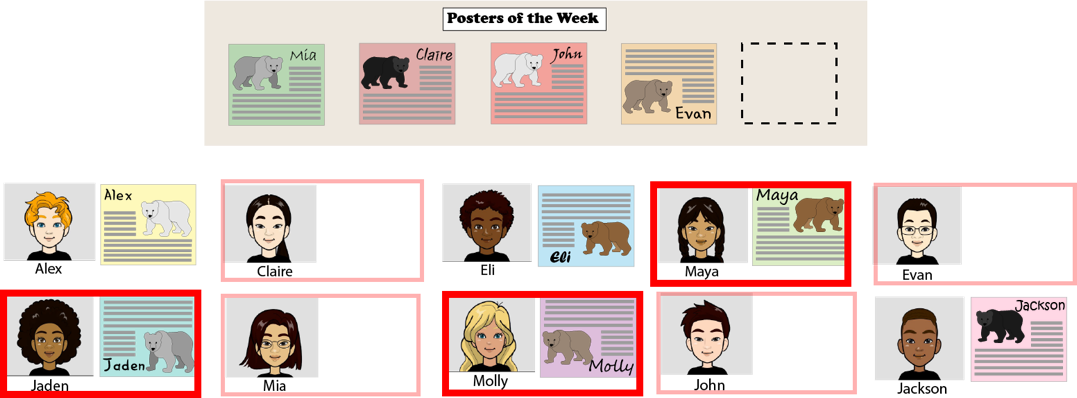


*[Initial Prediction Item]*

Who do you think Ms. Parker **will** pick to have their poster hung on the last spot on the wall? Click/circle to show who you think she will pick.

*[Reasoning]*

*Why* do you think Ms. Parker **will** choose that student?

*[Fairness Message]*

The next day, all the teachers in the school go to a meeting. At the meeting, a visitor talks about how it's fair to give all students a chance to do well, even if they look different. Sometimes teachers assume students who look different don't know as much. But it's important to give all students the same opportunities. Ms. Parker said the meeting was very good. The other teachers also said that they enjoyed the meeting and learned something new.

*[Manipulation Check]*

Who attended the meeting?

- All the teachers in the school, including Ms. Parker.
- Only some of the teachers in the school.

What did they learn at the meeting?

- It’s fair to give every student a chance, even if they look different.
- Students who look different don’t know as much.

Now it is the day after the meeting. Ms. Parker's class learned about desert plants today. In class, they make new posters about the plants they learned about. Everybody did a really good job!

Remember, Ms. Parker picked these students' posters **last time**:

***[****Repeated image of initial teacher preference, respective of condition.]*

Here are some other students in the class and their posters.

This time, Ms. Parker has room to hang **3 posters**. How **likely** is Ms. Parker to pick *these* students’ posters? *[Biased allocation]*

| Really not likely  O | Not likely  O | A little not likely  O | A little likely  O | Likely  O | Really likely  O |
| --- | --- | --- | --- | --- | --- |


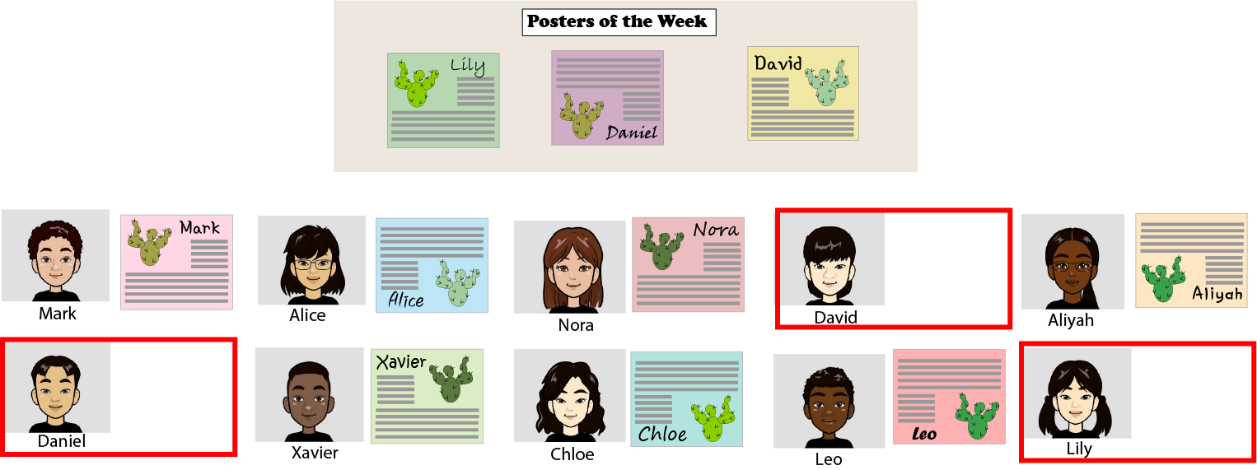


What if Ms. Parker picked **these** students' posters this time?

How **likely** is Ms. Parker to pick *these* students’ posters? *[Equal allocation]*

| Really not likely  O | Not likely  O | A little not likely  O | A little likely  O | Likely  O | Really likely  O |
| --- | --- | --- | --- | --- | --- |


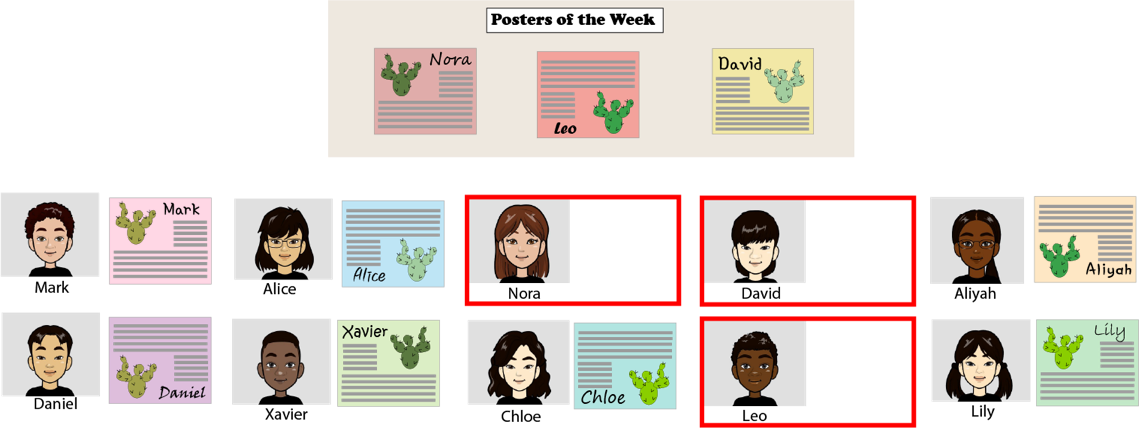


What if Ms. Parker picked **these** students' posters this time?

How **likely** is Ms. Parker to pick *these* students’ posters? *[Rectifying allocation]*

| Really not likely  O | Not likely  O | A little not likely  O | A little likely  O | Likely  O | Really likely  O |
| --- | --- | --- | --- | --- | --- |


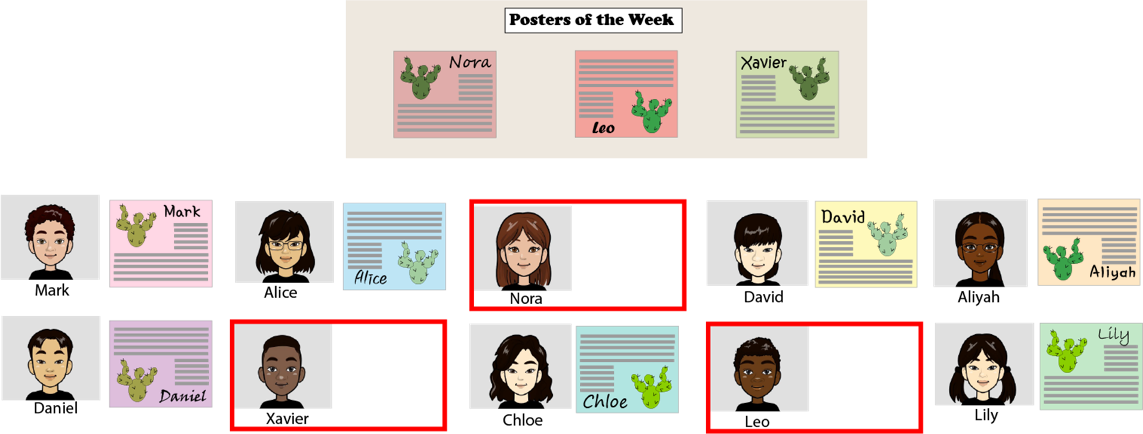


**Filler Items Between Vignettes:**

What type of snack is your favorite? Circle/click one. [*popcorn, ice cream, apply, pretzel]*

What activity is your favorite? Circle/click one. [*sports, reading, music, art*]

Great job! You’re about halfway done. Now we’re going to see another class.

**Peer-allocator vignette:**

Ms. Sanders’ class is another class at the school. Here are some of the students in Ms. Sanders’ class.

The students in Ms. Sanders’ class studied the planets in the solar system. They made posters about the planets they learned about. Here are some of the students and their posters. Everyone did a really good job!

The students really like it when their poster is put up on the wall. **Everyone hopes their poster will be chosen**.


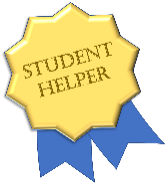


Ms. Sanders is not at school today. **Sam** is a student helper for the substitute teacher.

Sam is in charge of choosing 4 posters that will hang on the classroom wall.

These are the posters Sam picks to go on the wall.


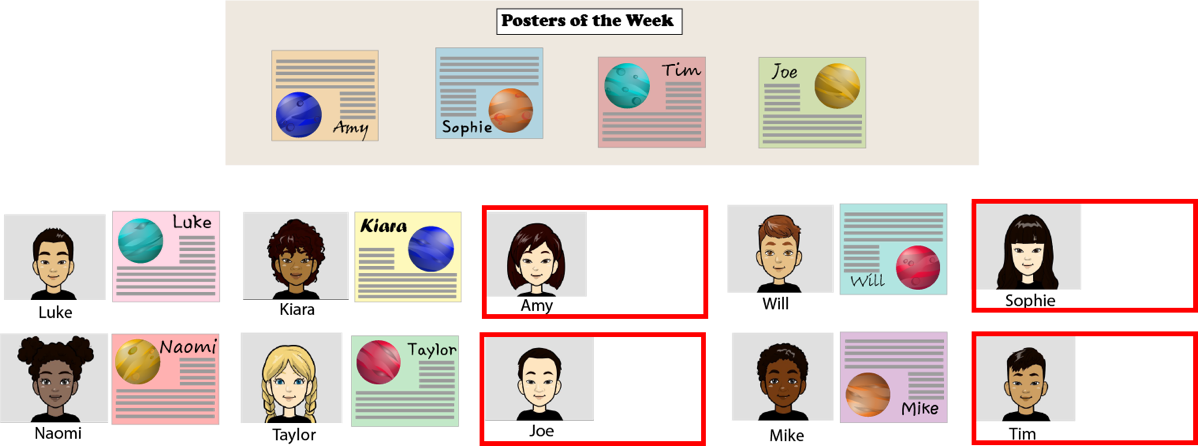


Later, Sam notices that there is room for one more poster on the classroom wall. Sam wants to hang one more poster, but can’t decide between **Luke, Will, and Mike’s** posters.


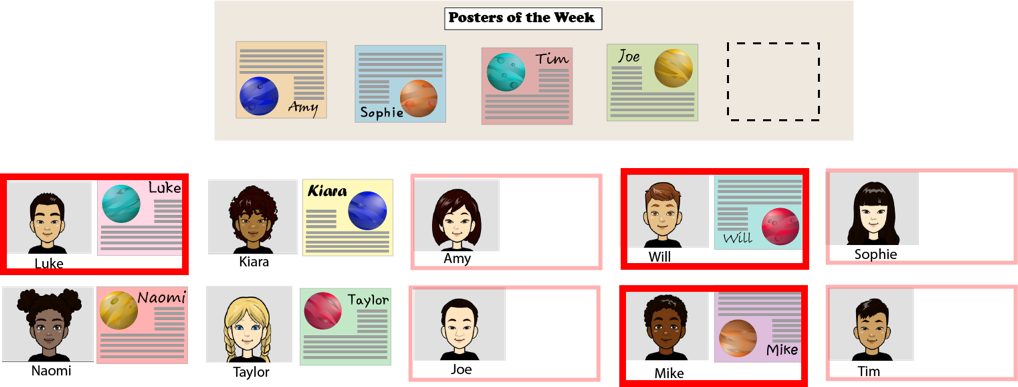


Later, Sam notices that there is room for one more poster on the classroom wall. Sam wants to hang one more poster, but can’t decide between **Luke, Will, and Jay’s** posters.

*[Initial Prediction Item]*

Who do you think Sam **will** pick to have their poster hung on the last spot on the wall? Click/circle to show who you think she will pick.

*[Reasoning]*

*Why* do you think Sam **will** choose that student?

*[Fairness Message]*

The next day, all the students in the school go to an assembly. Visitors talk about how it's fair to give everybody a chance, even if they look different. Some people assume that kids who look different don't know as much. But it's important to give everyone the same opportunities.

Sam said the assembly was very good. The other students also said that they enjoyed the assembly and learned something new.

*[Manipulation Check]*

Who attended the assembly?

- All the students in the school, including Sam.
- Only some of the students in the school.

What did they learn at the assembly?

- It’s fair to give everyone a chance, even if they look different.
- Kids who look different don’t know as much.

Now it is the day after the assembly. The students in Ms. Sanders’ class learned about birds of the rainforest today. In class, they make new posters about the birds they learned about. Everybody did a really good job!

The students really like it when their poster is put up on the wall. **Everyone hopes their poster is chosen.**


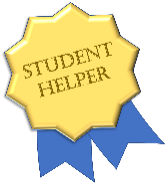


Ms. Sanders is not at school again today. **Sam** will be the student helper for the substitute teacher.

This time, Sam will choose **3 posters** to hang on the classroom wall.

Sam picked these students' posters **last time**. *[Repeated image of peer’s previous choice of four posters, respective to condition.]*

This time, Sam has room to hang **3 posters.** How **likely** is Sam to pick *these* students’ posters?

*[Biased allocation]*

| Really not likely  O | Not likely  O | A little not likely  O | A little likely  O | Likely  O | Really likely  O |
| --- | --- | --- | --- | --- | --- |


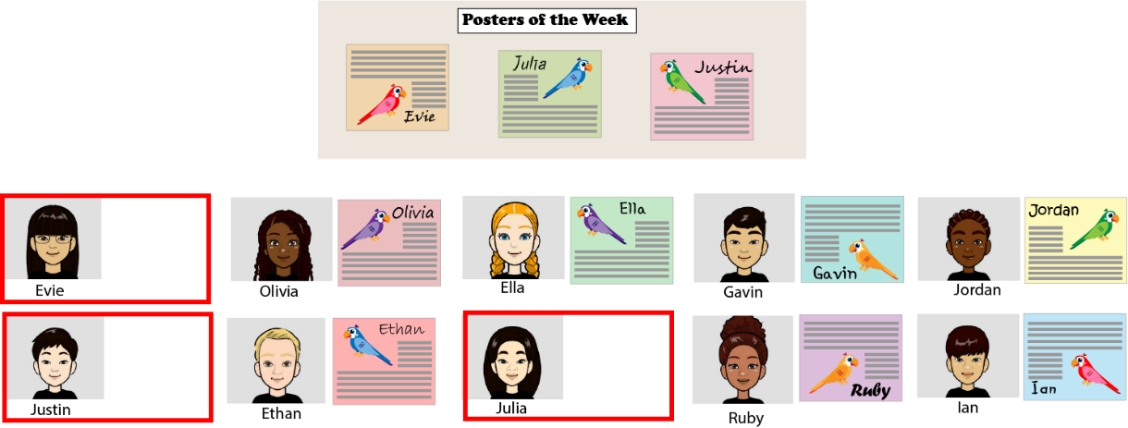


What if Sam picked **these** students' posters this time?

How **likely** is Sam to pick *these* students’ posters? *[Equal allocation]*

| Really not likely  O | Not likely  O | A little not likely  O | A little likely  O | Likely  O | Really likely  O |
| --- | --- | --- | --- | --- | --- |


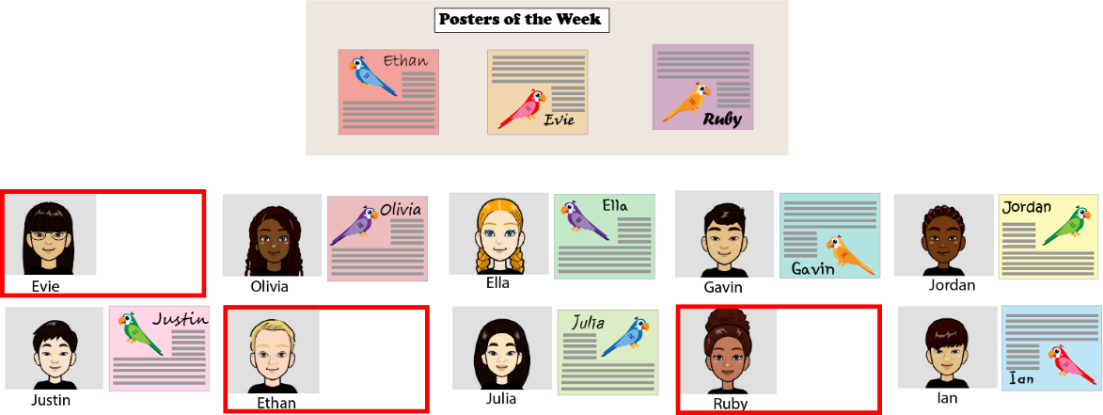


What if Sam picked **these** students' posters this time?

How **likely** is Sam to pick *these* students’ posters? *[Rectifying allocation]*

| Really not likely  O | Not likely  O | A little not likely  O | A little likely  O | Likely  O | Really likely  O |
| --- | --- | --- | --- | --- | --- |


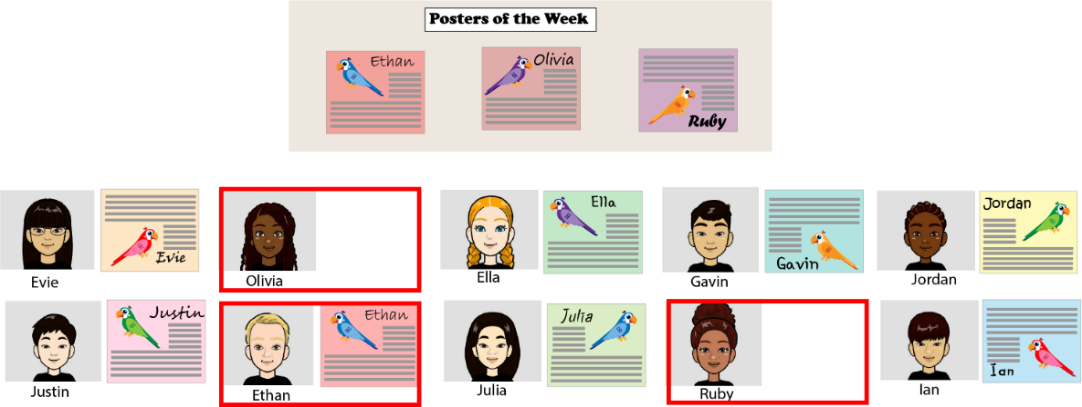


Great job! You're all done with the stories. Now we just have a few questions about you and what you think.

*[Perceived Numeric Racial Representation]*

Think about your **classes** at school. How many kids in your **class**(es) at school are the same **race** or ethnicity as you?

| None  O | A few  O | Some  O | A lot  O | All  O |
| --- | --- | --- | --- | --- |

Think about your **grade** at school. How many kids in your **grade** at school are the same **race** or ethnicity as you?

| None  O | A few  O | Some  O | A lot  O | All  O |
| --- | --- | --- | --- | --- |

Now think about your **whole school**. How many kids in your **school** are the same **race** or ethnicity as you?

| None  O | A few  O | Some  O | A lot  O | All  O |
| --- | --- | --- | --- | --- |

**Debrief**

Great job! To end the story, all the students in the school wrote book reports the next week.

In Ms. Sanders' class, Sam picked all the kids who had not had their posters on the wall.

In Ms. Parker's class, Ms. Parker also picked the kids who had not had their poster up.

In the end, all the students got a chance.

**Racial-ethnic identity items:**

What race or ethnicity are you? You can pick all that apply.

- Asian, Asian American (includes Filipino, other Pacific Islander, Chinese, Korean, Japanese, Indian, and more)
- Black or African American
- Latino or Hispanic (e.g. Mexican, Puerto Rican, Dominican)
- Middle Eastern or North African
- White or European American
- Other (write in)
- I don’t know.

Which race or ethnicity do you feel you identify with the *most*? This time, pick only one.

- Asian, Asian American (includes Filipino, other Pacific Islander, Chinese, Korean, Japanese, Indian, and more)
- Black or African American
- Latino or Hispanic (e.g. Mexican, Puerto Rican, Dominican)
- Middle Eastern or North African
- White or European American
- Other (write in)
- I don’t know.

**Reasoning Hypotheses, Analytic Approach, Results, and Interpretation**

Regarding participants’ reasoning for their expectations, we predicted that participants would use more reasoning recognizing bias about the teacher, but more personal choice reasoning about the peer, that adolescents would use more reasoning recognizing bias than would children, that participants viewing the allocator show a preference for Asian or White students would be more likely to use reasoning recognizing bias than those who viewed the allocator prefer Black students, and that reporting being in the ethnic-racial minority at their school would be associated with use of reasoning recognizing bias.

**Reasoning Measure.** Participants were asked, “Why do you think Ms. Parker/Sam will choose that student?” Participants provided open-ended responses explaining their reasoning.

**Categorically Coding Reasoning Responses.** Participants’ open-ended responses explaining their reasoning for their initial bias expectations were coded into conceptual categories for analysis. We developed the coding scheme based on prior literature (Cooley et al., 2019; Killen et al., 2024) as well as the social reasoning developmental model (Rutland et al., 2010). We examined a randomly selected set of 50 participant responses to refine the coding scheme so that codes were reflective of the themes in participant responses for this study. Coded reasoning categories were: (1) general fairness, (2) recognizing bias, (3) personal choice, (4) merit, (6) don’t know/uncodeable. See Table 1 for explanations and examples of each category.

The coding team consisted of three coders, who reached interrater reliability at Cohen’s 𝜅 > 0.90 and were blind to information about participants and to hypotheses at the time of coding. Each response was coded into a single category, as fewer than 10% of responses reflected multiple categories. Disagreement between coders was resolved through discussion (Åkerlind, 2005). Dummy variables for each reasoning type were then created to allow for quantitative analysis of hypotheses regarding the factors associated with participants’ use of specific reasoning categories, e.g., recognizing bias and personal choice.

**Table S3**

*Conceptual Categories Used to Code Reasoning for Expectation of Bias*

| Conceptual Category | Description | Participant Response Examples |
| --- | --- | --- |
| Recognizing bias | References to the racial preference, bias, diversity, or to the benefit of selecting typically underrepresented students | “Because everybody he chose was Black. So, since Jay is Black, I think he will choose him.”  “Because before she did not hang up any Black or Asian students.” |
| Personal choice | References to individual’s autonomy to choose according to their personal preference in allocating recognition, including friendship | “Maybe she likes her the best.”  “I think he will choose Daniel because that’s probably his friend.” |
| General Fairness | References to fair treatment toward all individuals, not referencing racial preference or bias | “Because he deserves this like everyone else.”  “Maybe because she didn’t get a chance last time.” |
| Merit | References to the effort of the selected students or to the quality of their academic work | “Because her poster is the most interesting and she put the most effort.”  “He did a nice job on the poster.” |
| “I don’t know” or Uncodeable | “I don’t know,” or otherwise indiscernible. | “I don’t know.”  “I guessed, honestly I have no clue.” |

*Note:* Coded reasoning categories and example participant justifications for responses to “Why do you think Ms. Parker/Sam will choose that student?”

To test our expectations about participant reasoning, we conducted separate generalized linear mixed effects models with a logit link predicting each hypothesized reasoning type, where an outcome of 1 = used reasoning category, 0 = used any other reasoning category. Predictors in each model were allocator (teacher/peer), age group, condition, participant race, and PNRR, with a random intercept for participant, to test for the allocator repeated measure.

**Results**

In analyzing our hypothesis regarding use of personal choice reasoning, fewer than 10% of participants used this reasoning type, resulting in biased conditional odds ratios. Participants were significantly more likely to use personal choice concerns (e.g., “It’s his choice, that’s just who he likes best”) in explaining their expectation of the peer (8% of participants) than in explaining their expectation of the teacher (2% of participants). Yet given the low frequency of this reasoning category, our planned generalized linear mixed model demonstrated very large conditional odds ratios (*OR*: 1652.96, 95% CI [165.46, 16513.78], *p* < .001). To account for this, we followed up with a population-averaged logistic regression using generalized estimating equations for bias correction, which also confirmed our hypothesis (*OR* = 5.42, 95% CI [2.45, 11.99], *p* < .001). As participants were more likely to reason about personal choice regarding a peer, it may be that some youth assumed that the peer’s allocation of academic recognition represented a friendship-based preference, rather than a racially biased one. This could be because youth have an easier time imagining the many possibilities of how a fellow student might behave, compared with a teacher.

Contrary to our hypothesis, participants did not use more reasoning recognizing bias about the teacher than about the peer. In separate follow-up logistic regressions for each vignette, we confirmed our expectation regarding the effect of age on reasoning, adolescents had higher log odds than children to recognize bias in their reasoning about both the peer’s (*OR*: 3.53, 95% CI [2.06, 6.17], *p* < .001) and teacher’s preference (*OR*: 2.96, 95% CI [1.73, 5.15], *p* < .001). We also did not find evidence for our hypotheses that use of reasoning recognizing bias would differ by condition or PNRR. While we did not find a difference between use of reasoning recognizing inequality regarding the teacher allocator and peer allocator, it is worth noting that youth reasoned referencing bias (29% of participants in both vignettes) far more frequently than they referenced personal choice (8% in peer vignette and 2% in teacher vignette). This indicates that youth were more likely to interpret the racial preference as representing a bias than to interpret it as merely a matter of individual personal preference, regardless of the teacher or peer allocator.

**Full Odds Ratio Results for Multinomial Logistic Regressions Predicting Initial Expectations (H1)**

In the **teacher vignette**, as shown in Figure 3, youth in the Asian-preferred condition were more likely than those in the Black-preferred condition to predict the teacher would choose another Asian student over a Black student (*OR*: 13.89, 95% CI [6.07, 31.78], *p* < .001) and were more likely than those in the White-preferred condition to predict the teacher would choose another Asian student over a White student (*OR*: 3.48, 95% CI [1.77, 6.82], *p* < .001). In the Black-preferred condition, participants were more likely than those in the White-preferred condition to expect the teacher to select a Black student to a White student (*OR*: 12.03, 95% CI [5.70, 25.41], *p* < .001) and were more likely than those in the Asian-preferred condition to expect the teacher to pick another Black student over an Asian student (*OR*: 13.89, 95% CI [6.07, 31.78], *p* < .001). Youth in the White-preferred condition were more likely than those in the Asian-preferred condition to expect the teacher to pick another White student over an Asian student (*OR*: 3.48, 95% CI [1.77, 6.82], *p* < .001) and were more likely than those in the Black-preferred condition to expect the teacher to pick a White over a Black student (*OR*: 12.03, 95% CI [5.70, 25.41], *p* < .001). Follow-up Holm-Bonferroni-adjusted pairwise condition contrasts on predicted probabilities demonstrated further confirmation of our hypothesis within the teacher vignette. Participants in the Asian-preferred condition were more likely than those in either the Black-preferred (*p* < .001) or White-preferred (*p* = .016) conditions to expect the teacher to choose an Asian student. Youth in the Black-preferred condition were more likely than those in either other condition (*p*s < .001) to expect the teacher to select another Black student, and youth in the White-preferred condition were more likely than those in both the Asian-preferred (*p* = .004) and Black-preferred (*p* < .001) conditions to expect the teacher to select a White student.

In the **peer vignette**, participants in the Asian-preferred condition were more likely than those in the Black-preferred condition to predict the peer would choose an Asian student over a Black student (*OR:* 2.28, 95% CI [1.17, 4.43], *p* = .015), and more likely than those in the White-preferred condition to predict the peer would choose an Asian student over a White student (*OR*: 2.75, 95% CI [1.31,  5.80], *p* = 0.008). Youth in the Black-preferred condition were more likely than those in the White-preferred condition to expect the peer to select a Black student to a White student (*OR*: 5.29, 95% CI [2.41, 11.60], *p* < .001). Participants who saw the White-preferred condition were more likely than those in the Asian-preferred condition to predict the peer would pick a White student over an Asian student (*OR*: 2.75, 95% CI [1.31, 5.80], *p* = 0.008) and were more likely than those in the Black-preferred condition to predict the peer to select a White over a Black student (*OR*: 5.29, 95% CI [2.41, 11.60], *p* < .001). In our follow-up pairwise condition contrasts on predicted probabilities for the peer vignette, we found a more complex role of condition in participants’ expectations. Participants in both the Asian-preferred condition (*p* = .013) and Black-preferred condition (*p* = .020) were more likely than those in the White-preferred condition to predict the peer would choose an Asian student, but those in the Asian-preferred condition were not significantly more likely than those in the Black-preferred condition to expect the peer to choose an Asian student. Participants in the Black-preferred condition were more likely to predict another Black student would be selected by the peer compared with those in the Asian-preferred condition (*p* = .002) but not compared with those in the White-preferred condition, after the Holm-Bonferroni correction (*p* = .107). Youth in the White-preferred condition were more likely to expect another White student to be selected by the peer compared with those in the Black-preferred condition (*p* < .001), though not compared with those in the Asian-preferred condition.

Unexpectedly, participants in the Asian-preferred condition were also more likely to predict a White student would be chosen than did those in the Black-preferred condition (*p* = .001). These follow-up comparisons add additional context to the findings from our multinomial logistic regression model for the peer allocator vignette, indicating that each condition did not function completely equally. Rather, in the Black-preferred condition, there was the clearest pattern of expectations of the peer maintaining their racial preference, while in the Asian-preferred condition, participants did not as strongly expect the peer to maintain the racial preference. See Figure 4.
